# Precise high-throughput online near-infrared spectroscopy assay to determine key cell wall features associated with sugarcane bagasse digestibility

**DOI:** 10.1186/s13068-021-01979-x

**Published:** 2021-05-29

**Authors:** Xinru Li, Fumin Ma, Chengping Liang, Maoyao Wang, Yan Zhang, Yufei Shen, Muhammad Adnan, Pan Lu, Muhammad Tahir Khan, Jiangfeng Huang, Muqing Zhang

**Affiliations:** 1 Guangxi Key Laboratory of Sugarcane Biology, Sugar Industry Collaborative Innovation Center, State Key Laboratory for Conservation and Utilization of Subtropical Agro-Bioresources, Guangxi University, Nanning, 530004 Guangxi China; 2 Sugarcane Biotechnology Group, Nuclear Institute of Agriculture (NIA), Tando Jam, Pakistan

**Keywords:** Sugarcane bagasse, Cell wall, Biomass digestibility, NIRS, Cellulose crystallinity, Lignin

## Abstract

**Background:**

Sugarcane is one of the most crucial energy crops that produces high yields of sugar and lignocellulose. The cellulose crystallinity index (CrI) and lignin are the two kinds of key cell wall features that account for lignocellulose saccharification. Therefore, high-throughput screening of sugarcane germplasm with excellent cell wall features is considered a promising strategy to enhance bagasse digestibility. Recently, there has been research to explore near-infrared spectroscopy (NIRS) assays for the characterization of the corresponding wall features. However, due to the technical barriers of the offline strategy, it is difficult to apply for high-throughput real-time analyses. This study was therefore initiated to develop a high-throughput online NIRS assay to rapidly detect cellulose crystallinity, lignin content, and their related proportions in sugarcane, aiming to provide an efficient and feasible method for sugarcane cell wall feature evaluation.

**Results:**

A total of 838 different sugarcane genotypes were collected at different growth stages during 2018 and 2019. A continuous variation distribution of the near-infrared spectrum was observed among these collections. Due to the very large diversity of CrI and lignin contents detected in the collected sugarcane samples, seven high-quality calibration models were developed through online NIRS calibration. All of the generated equations displayed coefficient of determination (*R*^2^) values greater than 0.8 and high ratio performance deviation (RPD) values of over 2.0 in calibration, internal cross-validation, and external validation. Remarkably, the equations for CrI and total lignin content exhibited RPD values as high as 2.56 and 2.55, respectively, indicating their excellent prediction capacity. An offline NIRS assay was also performed. Comparable calibration was observed between the offline and online NIRS analyses, suggesting that both strategies would be applicable to estimate cell wall characteristics. Nevertheless, as online NIRS assays offer tremendous advantages for large-scale real-time screening applications, it could be implied that they are a better option for high-throughput cell wall feature prediction.

**Conclusions:**

This study, as an initial attempt, explored an online NIRS assay for the high-throughput assessment of key cell wall features in terms of CrI, lignin content, and their proportion in sugarcane. Consistent and precise calibration results were obtained with NIRS modeling, insinuating this strategy as a reliable approach for the large-scale screening of promising sugarcane germplasm for cell wall structure improvement and beyond.

**Supplementary Information:**

The online version contains supplementary material available at 10.1186/s13068-021-01979-x.

## Background

Bioethanol has been recognized as a significant clean fuel to reduce carbon debt. In particular, cellulosic ethanol derived from lignocellulosic feedstock has received increasing attention because it does not compete with food production or occupy the land otherwise used for this purpose [1]. Sugarcane is one of the essential sugar and energy crops worldwide. In particular, bagasse, a significant byproduct of sugarcane crushing during juice extraction, shows great advantages for second-generation biofuel production [2]. However, due to cell wall recalcitrance to hydrolysis, the cost-effectiveness of cellulosic ethanol production from sugarcane remains in question [3]. Therefore, screening germplasm for optimal cell wall features is vital for the use of sugarcane as a biofuel crop.

Plant cell walls are composed of three different polymers, i.e., cellulose, hemicellulose, and lignin. These polymers form a complex network structure that impedes cell wall digestibility [4]. The properties of cellulose and lignin are mainly related to cell wall recalcitrance [5–8]. For instance, cellulose is a polymer composed of glucose units linked via β-1,4-glycosidic bonds. The cellulose crystallinity index (CrI), which is characterized by X-ray scattering from crystalline and amorphous regions [9, 10], is a critical parameter that defines hindrance to cell wall saccharification [5, 8, 11, 12]. Lignin is a hydrophobic polymer composed of phenylpropane compounds that often tightly associated with hemicellulose to form lignin–carbohydrate complexes (LCCs). This “LCC complex” blocks the cellulose surface and hinders cellulose accessibility [13]. Therefore, lignin is also a significant factor that affects cell wall saccharification [6, 14, 15]. Thus, the screening of germplasm resources for lower cellulose CrI and lignin content can play a significant role in modifying cell wall recalcitrance attributes. A high-throughput assay is urgently needed to determine cell wall characteristics.

Near-infrared spectroscopy (NIRS) is a rapid and nondestructive analytical tool for high-throughput biomass quantity or quality analysis for biofuel production [16]. It has been used to characterize cell wall polymer features [17–20], analyze biomass saccharification efficiency [17, 18, 21], and predict ethanol production via yeast fermentation [22–25]. Notably, in sugarcane, some studies have also applied NIRS for determining cell wall components or predicting digestibility [26–29]. In one such effort, Caliari et al. [30] explored an NIRS assay to estimate the cellulose crystallinity index. However, most studies have used an offline calibration strategy that necessitates specific time-consuming NIRS scanning steps. Hence, they are limited in their analysis of many samples, which are generally required in crop improvement programs.

This study was initiated to develop a high-throughput online NIRS assay to characterize key cell wall features in sugarcane bagasse. Hundreds of samples were collected from the sugarcane germplasm. Based on the standard laboratory analytical methods for cell wall features and the online system for near-infrared spectroscopy, a reliable online NIRS assay was developed for analyzing the lignin content and CrI. Thus, this study provides a precise and high-throughput approach for large-scale screening and selection of optimal germplasm to reduce cell wall recalcitrance and target genetic improvements in sugarcane for low-cost bioethanol production.

## Results

### Near-infrared spectroscopy-based characterization of collected sugarcane samples

**Fig. 1:**
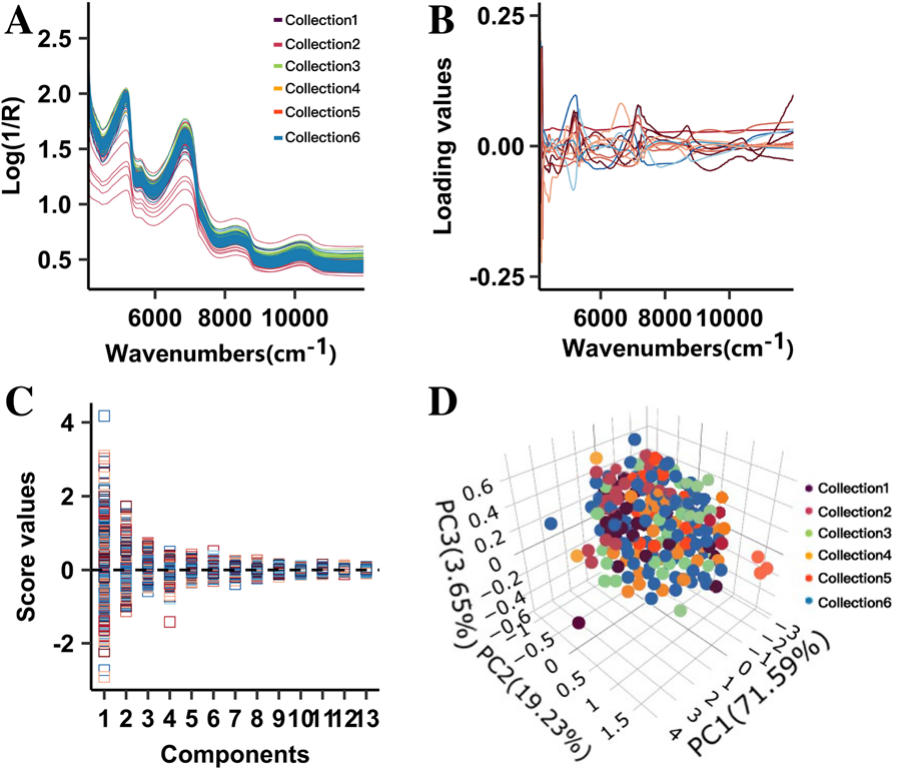
Near-infrared spectral characterizations in the sugarcane population. **A** Original spectroscopy. **B** The first 13 principal components for near-infrared spectral characterization. **C** Sample variations in each principal component in the sugarcane samples. **D** 3D view of the collected sugarcane samples via PCA

A total of 838 germplasm samples collected in six different batches were used for online NIRS modeling (Additional file 1: Table S1). While analyzing each sample lot, the NIRS data were immediately collected on an explicitly designed online system. The continuously collected spectrum reflectance values were automatically averaged for NIRS calibration by OPUS software (build: 7.8.44, Bruker Optik GmbH 2016). As shown in Fig. 1A, the near-infrared spectral reflectance values of all samples displayed a fluctuation within the normal range, indicating the diverse nature of these samples. Principal component analysis (PCA) was carried out to characterize the distribution of these collected samples from the recorded near-infrared spectral values. In PCA, new orthogonal variables were generated from the original spectral values. The first 13 principal components (PCs), which could explain 99.81% of the variation, were selected to characterize the sample distribution (Fig. 1B). Considerable variations in the collected samples were observed within the selected PCs, especially for the first five PCs (Fig. 1C). Finally, the first three PCs were used for a 3D observation of the sample distribution. Although sugarcane samples of different genotypes were collected from different batches, no discriminable distribution was observed among them (Fig. 1D), suggesting that these samples could be exploited for global NIRS modeling.

## Diversity of cell wall features in the collected sugarcane samples

**Fig. 2:**
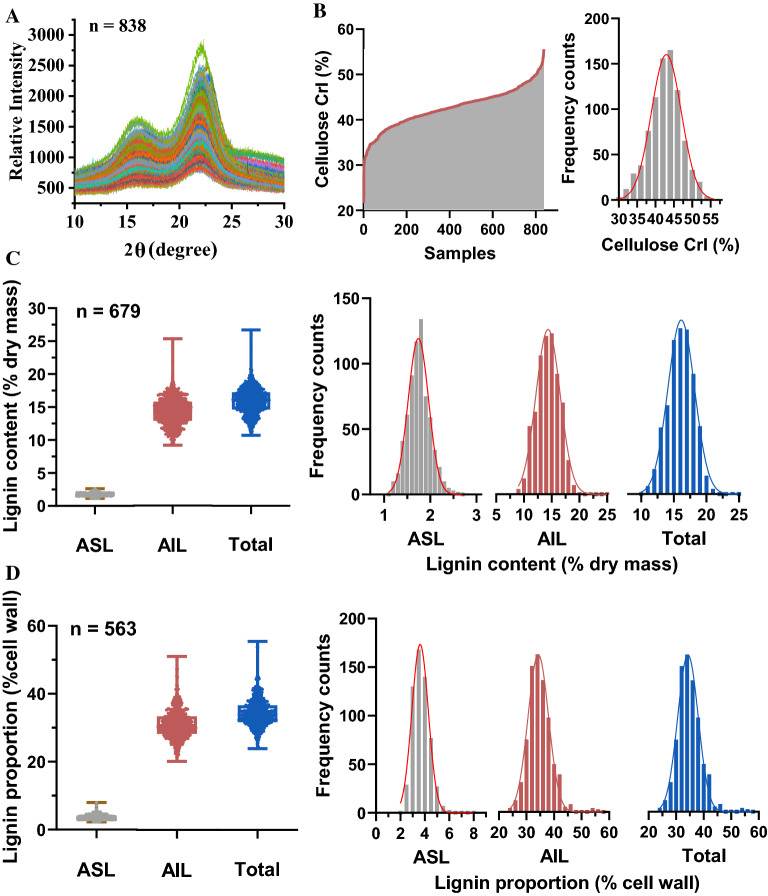
Diversity of cell wall features in the sugarcane population. **A** X-ray diffractograms. **B** Distribution and variations in cellulose crystallinity. **C** Variation in lignin content (% dry mass) in the collected sugarcane samples. **D** Variation in lignin proportion (% cell wall) in the collected sugarcane samples. *ASL* acid-soluble lignin; *AIL* acid-insoluble lignin

X-ray diffraction (XRD) was applied for cellulose CrI determination. The maximum and minimum diffraction were separately observed in the 2θ region ranging from 15° to 25°, allowing for a standard calculation of CrI. Various diffraction values were observed in the collected sugarcane samples, depicting genotype diversity (Fig. 2A). The maximum and minimum diffraction values were applied for cellulose CrI calculation. The CrI calculated ranged from 21.6 to 55.6% (Fig. 2B; Additional file 1: Table S2), which is comparable with previous reports for sugarcane and *Miscanthus* [8, 30]. Moreover, the statistical distribution showed that cellulose CrI exhibited a normal distribution in the analyzed sugarcane collections (Fig. 2B). The diversity of the CrI values indicated considerable variation in cellulose-related features in the samples.

Lignin content (% dry mass) was analyzed through a two-step acid hydrolysis process combined with ashing. The acid-soluble lignin (ASL), acid-insoluble lignin (AIL), and the total of the two were determined. The ASL content (% dry mass) varied from 1.2 to 2.6%, while the AIL ranged from 9.2 to 25.3%. Moreover, a large variation in the total lignin content (% dry mass), which ranged from 10.9 to 27.0%, was observed (Fig. 2C; Additional file 1: Table S2). As lignin is closely related to the cell wall network structure that significantly impacts lignocellulose digestibility, this study also estimated the lignin proportion in the sugarcane cell wall. The lignin proportion exhibited a greater variation, especially for the ASL values, which illustrated the highest coefficient of variation (CV) of 0.19 (Fig. 2D; Additional file 1: Table S2). The total lignin proportion (% cell wall) ranged from 24.3 to 56.2%, depicting a variation of the cell wall structure in the collected sugarcane samples. Furthermore, a normal distribution was observed in both the lignin content and proportion (Fig. 2C, D), suggesting a reliable NIRS calibration.

## Characterization of the calibration and validation sets

**Fig. 3:**
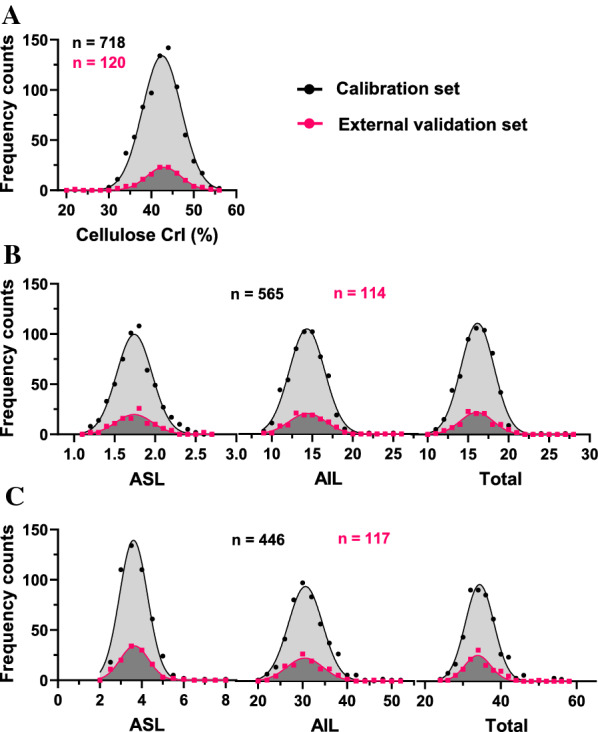
Sample distribution in the calibration and validation sets for online NIRS modeling. **A** Cellulose crystallinity. **B** Lignin clean mass content in the dry biomass. **C** Lignin proportion in the cell wall. *ASL* acid-soluble lignin; *AIL* acid-insoluble lignin

The samples were divided into two sets for online NIRS modeling and the following performance evaluation: one set for NIRS calibration and another for external validation. For cellulose CrI modeling, a total of 120 samples were randomly selected from the sample population to build an external validation set, and the remaining 718 samples formed the calibration set (Fig. 3A). Similarly, a total of 679 samples were used for lignin content (% dry mass) modeling, 565 for calibration, and 114 for external validation (Fig. 3B). For lignin proportion, 446 and 117 samples were analyzed for calibration and equation evaluation, respectively (Fig. 3C). Moreover, a frequency distribution was carried out to compare the calibration and validation sets of the cell wall features. Notably, all of these values were comparable and showed a similar normal distribution (Fig. 3A–C). Hence, these comparable data sets allowed reliable online NIRS modeling and external validation.

## Online NIRS modeling

Partial least square (PLS) regression analysis methods contained in OPUS software according to the “Setup Quant 2 method” module was performed for NIRS modeling. Dozens of parameters were combined in terms of wavelength range selection and spectrum pretreatment to obtain calibration equations in PLS analysis. Internal cross-validation or external validation was carried out to evaluate the performance of the equations, and then the best equations were obtained (according to their high-performance invalidation).

**Fig. 4:**
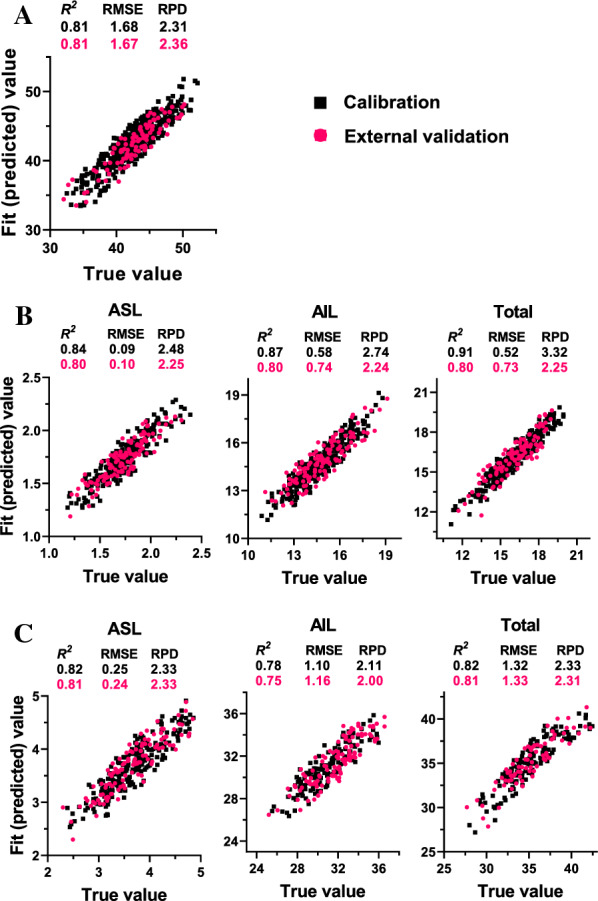
Equation performance during online NIRS calibration and external validation. **A** Cellulose crystallinity. **B** Lignin clean mass content in the dry biomass. **C** Lignin proportion in the cell wall. *ASL* acid-soluble lignin; *AIL* acid-insoluble lignin; *RMSE* root mean square error; *RPD* ratio performance deviation

The calibration results showed that all of the equations produced for the two cell wall features exhibited high coefficient of determination (*R*^2^) values, over 0.80, except for the AIL proportion, which showed an *R*^2^ value of 0.78 (Fig. 4; Table 1). The total lignin content equation from the dry biomass determination demonstrated the highest fit performance, with the maximum observed *R*^2^ value of 0.91 (Fig. 4B; Table 1). Therefore, the identified excellent correlations between the fit and reference values during calibration indicated the high prediction capacity of the obtained equations.

**Table 1:** Statistics for calibration and external validation parameters of the equations generated for cell wall feature prediction in sugarcane stalks

	Calibration									External validation			
	Rank	N	SCM	Spectrum range (cm^−1^)	Mean	SD	*R*^2^	RMSEC	RPD	N	*R*^2^ev	RMSEP	RPD
Cellulose Crl	24	403	SNV	11,964.8 – 11,178; 8825.1 – 7243.7; 6464.5 – 4104	42.68	3.75	0.81	0.68	2.31	97	0.81	1.67	2.36
Lignin content (% dry mass)													
ASL	8	254	SSL	11,964.8 – 10,391.1; 8825.1 – 7243.7	1.74	0.23	0.84	0.09	2.48	122	0.8	0.1	2.25
AIL	12	257	SNV	9612 – 7251.4; 6464.5 – 5677.7	14.75	1.55	0.87	0.58	2.74	128	0.8	0.74	2.24
Total	8	214	FD + SSL	9411.4 – 5446.3	16.45	1.54	0.91	0.52	3.32	101	0.8	0.73	2.25
Lignin content (% dry mass)													
ASL	18	222	FD + SNV	11,185.7 – 10,391.1; 9612 – 7243.7; 6464.5 – 5670; 4890.8 – 4104	3.67	2.26	0.82	0.25	2.33	111	0.81	0.24	2.33
AIL	7	144	SNV	9411.4 – 8886.8; 7868.5 – 7344; 5801.1 – 5276.6; 4767.4 – 4242.8	30.95	0.55	0.78	1.1	2.11	73	0.75	1.16	2
Total	6	147	FD + SNV	5785.7 – 5446.3	35.12	3	0.82	1.32	2.33	74	0.81	1.33	2.31

*ASL* acid-soluble lignin; *AIL* acid-insoluble lignin; *N* sample number; *SCM* scatter correction methods; *SD* standard deviation of reference value; *R*^2^ determination coefficient; *RMSEC* root mean square error of calibration; *RMSEP* root mean square error of external validation; *R*^2^ev determination coefficient of external validation; *RPD* ratio performance deviation; *SNV* standard normal variate; *SSL* straight-line subtraction; *FD + SSL* a combinations of first derivative and straight-line subtraction; *FD + SNV* a combination of FD and SNV

In addition, the samples from the external validation sets were applied as an independent validation assay to evaluate the prediction performance of the obtained models. Correlation analysis between the predicted values and the measured values was carried out, and the root mean squared error of prediction (RMSEP) and ratio performance deviation (RPD) were calculated. The results suggested that all of the equations exhibited a high correlation between the predicted and true values. The determination coefficient of external validation (*R*^2^ev) ranged from 0.75 to 0.81 (Fig. 4; Table 1). The AIL proportion showed an* R*^2^ev value of 0.75, which was consistent with the calibration results. Notably, all of the equations gave RPD values higher than 2.0 during external validation, suggesting their excellent prediction performance.

Finally, to achieve better performance of the equations for cell wall feature prediction, samples in the external validation set were combined into the global NIRS modeling calibration. As more samples were added, a wider variety of cell wall features was observed in the integrated new calibration sets (Table 2). As expected, most of the equations demonstrated substantial improvement in prediction capacity. In detail, the equation for cellulose CrI prediction showed the most remarkable improvement, as its *R*^2^ value rose from 0.81 to 0.88 (Table 2; Additional file 1: Figure S1A). AIL exhibited the maximum amelioration for lignin content and proportion prediction (Table 2; Additional file 1: Figure S1B, C). The new equations obtained from this analysis showed a high correlation between fit and the measured values, suggesting their excellent fitting during calibration. During cross-validation, the calibration set was randomly partitioned into several groups, and samples in each group were validated using a calibration equation developed from other samples. The results suggested that all of the generated equations exhibited high *R*^2^cv and RPD values, especially RPD values, ranging from 2.21 to 2.56 (Table 2; Additional file 1: Figure S1D–F). The equations for lignin content (% dry mass) and cellulose CrI displayed the highest *R*^2^cv value of 0.85 (Table 2; Additional file 1: Figure S1E), hinting at consistency with the calibration results. Notably, the AIL proportion illustrated consistent and high *R*^2^ and *R*^2^cv values of 0.80 for calibration and validation, illustrating their stable prediction capacity (Table 2; Additional file 1: Figure S1F). Taken together, all of the newly generated equations demonstrated good *R*^2^, *R*^2^cv, and RPD values of calibration and internal cross-validation. Hence, the generated equations could be applied to determine cell wall features.

**Table 2:** Statistics for optimized equations generated for prediction of cell wall features in sugarcane bagasse

	Calibration								Cross-validation		
	Rank	N	SCM	Spectrum range (cm^−1^)	Mean	SD	RMSEC	*R*^2^	RMSECV	*R*^2^cv	RPD
Cellulose Crl	12	433	FD	11,972 – 11,185.1; 10,398.3 – 7251.1; 5677.4 – 4890.6	42.8	3.85	1.37	0.88	1.5	0.85	2.56
Lignin content (% dry mass)											
ASL	8	380	FD + MSC	11,972.5 – 10,398.8; 8825.1 – 8038.3; 6464.5 – 5677.7	1.73	2.44	0.1	0.83	0.1	0.8	2.24
AIL	14	387	MMN	9612 – 8038.3; 6464.5 – 5677.7	14.8	2.95	0.55	0.89	0.7	0.8	2.26
Total	14	408	SNV	11,185.1 – 10,398.3; 9611.5 – 7251.1	16.15	1.67	0.51	0.91	0.66	0.85	2.55
Lignin proportion (% cell wall)											
ASL	8	333	FD	11,972.5 – 11,185.7; 10,398.8 – 9612; 8825.1 – 8038.3; 6464.5 – 5677.7	3.67	2.4	0.24	0.83	0.25	0.8	2.25
AIL	1	182	SNV	7251.4 – 5677.7	30.94	2.16	0.98	0.8	0.97	0.8	2.25
Total	6	221	FD + SNV	5785.7 – 5446.3	35.1	2.32	1.32	0.81	1.35	0.8	2.24

*ASL* acid-soluble lignin; *AIL* acid-insoluble lignin; *N* sample number; *SCM* scatter correction methods; *SD* standard deviation of reference value; *RMSEC* root mean square error of calibration; *R*^2^ determination coefficient; *RMSECV* root mean square err of cross-validation; *R*^2^*cv* determination coefficient of cross-validation; *RPD* ratio performance deviation; *FD* first derivative; *SNV* standard normal variate; *MSC* multiplicative scattering correction; *MMN* Min–Max normalization; *FD + SNV* a combination of FD and SNV; *FD + MSC* a combination of FD and MSC

## Offline NIRS modeling

**Fig. 5:**
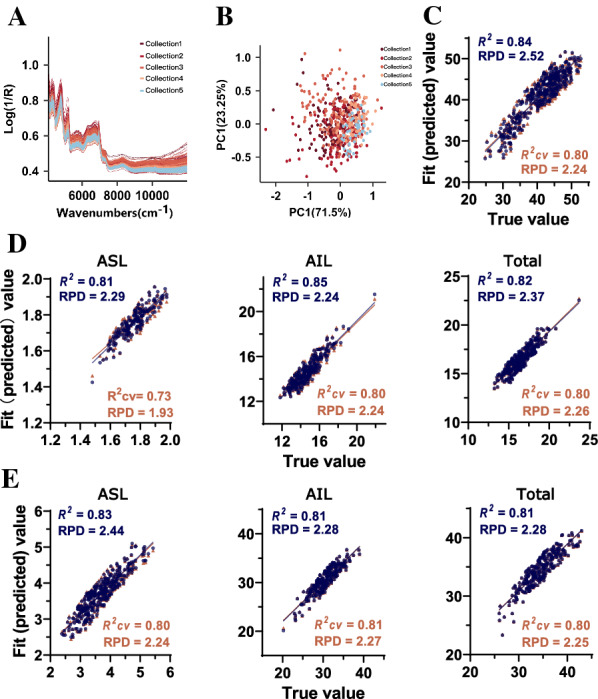
Equation prediction capacity of the offline NIRS models. **A** Original spectra of the dry sugarcane samples. **B** Principal component analysis of the samples in the calibration sets. **C** Calibration model of cellulose crystallinity, **D** lignin clean mass content in the dry biomass, and **E** lignin proportion in the cell wall. *ASL* acid-soluble lignin; *AIL* acid-insoluble lignin; *RPD* ratio performance deviation

As a comparison, this study also applied an offline NIRS calibration for these two kinds of key cell wall features. A total of 628 samples (the first five collections) were taken for offline NIRS modeling. Shredded fresh samples were dried and ground for offline NIR spectral data collection. As shown in Fig. 5A, the offline collected NIR spectra exhibited a different pattern from the online spectra. PCA showed that all of the sugarcane collections from different batches displayed continuous distribution (Fig. 5B), permitting reliable NIRS calibration.

PLS regression analysis was performed for offline NIRS calibration, and internal cross-validation was carried out to evaluate the performance of the equations. The calibration results showed that cellulose CrI exhibited the high *R*^2^ and RPD values of 0.84 and 2.52, respectively (Fig. 5C). For lignin content (% dry matter) modeling, the equations were observed with high *R*^2^ values ranging from 0.81 to 0.85, and AIL exhibited the most relevant results (Fig. 5D). Lignin proportion calibration also exhibited perfect fitting results. All of the obtained equations showed a high *R*^2^ value over 0.80 (Fig. 5E). Consequently, most of the equations displayed high *R*^2^cv and RPD values, except for the equation for acid-soluble lignin content (% dry matter), which maintained the low *R*^2^cv value of 0.73 (Fig. 5C–E).

## Discussion

The plant cell wall structure governs the biomass digestibility. In particular, lignin content and cellulose CrI are the two key features that dominantly hinder the utilization of cellulose in second-generation ethanol production [31, 32]. To reduce cell wall recalcitrance, attempts have been made to modify the cell wall structure by reducing cellulose crystallinity and lignin content in sugarcane [33–36] and other energy plants [12, 37–40]. Transgenic plants engineered to aim for desired variations in these characteristics have shown significant improvement in cell wall saccharification. Therefore, these cell wall features should be the traits of interest for energy cane breeding. Association studies through large-scale phenotypic and genotypic analyses have emerged as a promising strategy for crop improvement.

For the precise evaluation of crop genotypes and reliable germplasm selection, samples should be analyzed as soon as possible after collection. The considerable number of samples in such screening jobs necessitates the use of appropriate high-throughput techniques. However, due to the lack of effective phenotyping methods, it is difficult to obtain accurate phenotypic data. Some recent studies have explored offline NIRS assays for cellulose CrI and some other cell wall content determinations from sugarcane [26–29] due to technical shortages that limit their application in real-time online analysis. Therefore, it is imperative to establish a high-throughput online method to accurately evaluate key cell wall features in sugarcane.

This study reported an online NIRS assay for the high-throughput screening of the two key cell wall features described above. Hundreds of sugarcane genotypes were collected to obtain a sample set with wide variation for precise modeling in different ripening stages (Additional file 1: Table S1). As expected, considerable variation was observed in either the NIR spectra or these two kinds of cell wall features (Figs. 1, 2). Therefore, these normally distributed samples allow for reliable NIRS calibration. The produced equations showed high *R*^2^/*R*^2^cv/*R*^2^ev values of calibration, internal cross-validation, and external validation (Fig. 4; Additional file 1: Figure S1; Table 1), suggesting their high-quality performance.

Moreover, an offline NIRS calibration was also conducted for cell wall feature prediction and high *R*^2^ and RPD values were obtained for both cellulose CrI and lignin content. All of the equations exhibited a high linear correlation between the predicted and reference values during internal cross-validation (Fig. 5), suggesting their consistent prediction capacity. Notably, some of the offline NIRS models achieved better prediction performance than those reported previously [26, 30], which could be attributed to the large population of diverse samples employed for NIRS modeling in this study.

**Fig. 6:**
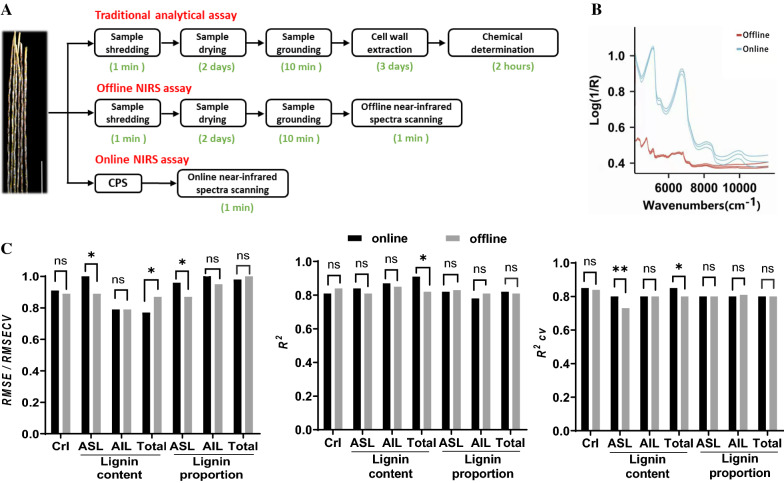
Comparison between the online and offline NIRS assays. **A** Proceedings of the online and offline NIRS analyses. **B** Near-infrared spectra collected during the online and offline procedures. **C** Statistical comparison of the model parameters between the offline and online NIRS assays. * and ** indicated statistically significant different at *p*  <  0.05 and 0.01, respectively

Additionally, these two different NIRS modeling strategies were compared in terms of their technical schedule and prediction performance. As shown in Fig. 6A, due to the cooperation of CPS and the online spectra scanning system, the online NIRS analysis could be completed within one minute. Therefore, it could be applied as a real-time online detection system for sugarcane cell wall feature determination. In contrast to the online methods, because more pretreatment steps are required prior to NIR spectrum collection, the offline NIRS methods were more time-consuming (Fig. 6A). Additionally, because the offline NIR spectra were collected based on the ground dry samples, they showed a different online pattern (Fig. 6B), which is consistent with previous offline NIRS studies in sugarcane and certain other crops [24, 26, 30]. More importantly, the calibration results showed that most of the obtained online equations exhibited comparable performance to the offline equations, and some of them even illustrated higher *R*^2^ and RPD values in both calibration and validation (Table 1; Additional file 1: Figure S1; Fig. 5). In addition, the equation performance was compared between the two different NIRS strategies. As a result, for most of the equations, no statistically significant differences in the RMSEC/RMSECV, *R*^2^ or *R*^2^cv values were detected between the online and offline NIRS assays (Fig. 6C). These results suggested that the online NIRS assay showed comparable or even better prediction capacity than the offline assay. Therefore, the online NIRS assay showed more advantages for large-scale screening jobs via a high-throughput real-time online detection system that could be considered a permissible strategy for sugarcane germplasm screening.

Taken together, this study explored both offline and online NIRS modeling to predict two kinds of crucial cell wall features that account for sugarcane bagasse digestibility. All of the equations produced in this research exhibited high prediction performance, suggesting their excellent potential for use in germplasm screening. Due to significant advantages in their protocols, the online calibration models developed in this study exhibit excellent prospects for the high-throughput screening of large-scale samples for energy cane breeding and germplasm selection.

## Conclusions

This study developed an online NIRS assay for the high-throughput analysis of crucial cell wall features in cellulose crystallinity, lignin content, and their proportion in sugarcane. Because vast amounts of varied sugarcane samples were applied for NIRS calibration, consistent and precise modeling results were observed with high *R*^2^, *R*^2^cv, *R*^2^ev, and RPD values, exhibiting their perfect prediction capacity. Most of these obtained online NIRS equations showed comparable or even much better performance than the offline equations. More importantly, the online detection system exhibited a much greater time efficiency. This allowed for real-time online analysis and demonstrated that the online NIRS strategy would be a reliable approach for a large-scale screening of optimal sugarcane germplasm. Therefore, this study provided a feasible solution for high-throughput screening jobs in energy cane breeding and beyond.

## Methods

### Sample collection

A total of 838 sugarcane germplasm was planted at the Fusui experimental field of Guangxi University. Hundreds of them were harvested at different growth stages in the year 2018 and 2019. In detail, 164, 162, 184, 70, and 48 samples were collected once a month from November 2018 to March 2019; moreover, 210 samples were collected in December 2019 (Additional file 1: Table S1). Six stalks were randomly selected for each genotype, and further analysis was carried out after removing leaves and young tips.

### Near-infrared spectral data collection

Online near-infrared spectral data collection: the selected six stalks of each genotype were shredded using DM540 (IRBI Machines & Equipment Ltd, Brazil). The shredded fresh samples were immediately blended and transferred for NIRS scanning by CPS (Cane presentation system, Bruker Optik GmbH, Germany). Near-infrared spectral data of fresh samples were simultaneously collected through MATRIX-F (Bruker Optik GmbH, Germany) online system.

Offline near-infrared spectral data collection: following the online NIR spectral data collection, the shredded samples were inactivated at 100 °C for 1 h and then dried under 60 ℃ until there was no loss of weight. The dried sample was ground through a 40-mesh screen and stored in a dry container until use. MATRIX-F, equipped with a Q413 sensor head, was used for offline NIR spectral data collection. Each sample was scanned three times.

Full-band scanning mode with the wavelengths ranging from 4000 to 10,000 cm^−1^ with 4 cm^−1^ steps was employed for collecting online and offline spectral data. The spectral absorbance values were recorded as log1/R, where R is the sample reflectance. A standard equipped in Matrix-F was scanned every one hour for instrument correction to ensure consistency of measurements. The obtained online reflectance values were automatically averaged by OPUS software, and the three replicates offline spectrums were manually averaged for further analysis.

### Lignin content determination

According to the National Renewable Energy Laboratory’s analytical procedure, a two-step acid hydrolysis was carried out to determine the lignin content with minor modification [41]. Briefly, 0.50 g of dry ground samples were extracted using benzene–ethanol (2:1, v/v) in a Soxhlet for 4 h and then hydrolyzed using 10.0 mL 67% (v/v) H_2_SO_4_ (at 25 °C for 90 min with gentle shaking at 115 r/min). After hydrolysis, the acid solution was subsequently diluted to 3.97% (w/w) with distilled water and heated at 115 °C for 60 min. The autoclaved hydrolysis solution was filtered through a filtering crucible. The supernatant liquids were fixed to 250 mL and read at 205 nm under UV spectroscopy to estimate acid-soluble lignin. The remaining residues were ashed in a muffle furnace at 575 °C ± 25 °C for 4 h to ascertain the acid-insoluble lignin [17, 21]. All experiments were conducted in triplicate.

Lignin proportion was determined according to the calculated lignin content in the cell wall. Briefly, 0.10 g of dry ground samples was extracted with water (at 50 °C for 2 h with shaking at 150 r/min) for total sugar content determination [42]. The residues were estimated as cell walls by subtracting soluble sugar from dry biomass. Finally, lignin proportion was calculated by dividing lignin content by cell wall.

### Lignocellulose crystallinity index determination

X-ray diffraction (XRD) method was used to determine lignocellulose crystallinity index (CrI) as described by Zhang et al. [8]. In detail, approximately 0.3 g of the ground dry samples were extracted using 10 mL of distilled water to remove the soluble sugar. The residues were subsequently extracted using chloroform–methanol (1:1, v/v), methanol, and acetone and then dried under vacuum conditions. The remaining residues were classified as crude cell walls and were used for examination through XRD.

Rigaku-D/MAX 2500 V instrument (Ultima III, Japan) was employed for XRD analysis. The crude cell wall powder was laid on the glass holder and investigated under plateau conditions. Ni-filtered Cu-Ka radiation (*k* = 0.154056 nm) generated at 40 kV voltage and 18 mA was used for this analysis. A continuous scanning from 10° to 45° was performed at the speed of 0.0197°/s. The CrI was estimated in terms of percentage by calculating the intensity of the 200 peaks (*I*_200_, *h* = 22.5°) and the intensity at the minimum between the 200 and 110 peaks (*I*_am_, *h* = 18.5°) as follows: ${\text{CrI}} = 100 \times \left( {I_{{{200}}} - I_{{{\text{am}}}} } \right)/I_{200}$where *I*_200_ represents both crystalline and amorphous materials, while *I*_am_ denotes amorphous material [43].

### NIRS data processing and calibration

The OPUS spectroscopy software (version 7.8, Bruker Optik GmbH, Germany) was used for data processing and NIRS calibration. To solve the problems associated with the overlapping peaks and baseline correction, pretreatment and the wavelength range selection of the raw spectral data were performed before calibration. Several spectral pretreatment methods were used in OPUS software, namely constant offset elimination (COE), straight-line subtraction (SSL), standard normal variate (SNV), Min–Max normalization (MMN), multiplicative scattering correction (MSC), first derivative (FD), second derivative (SED), a combination of the first derivative and straight-line subtraction (FD + SSL), a combination of the first derivative and standard normal variate (FD + SNV), and a combination of the first derivative and multiplicative scattering correction (FD + MSC). The NIRS spectra were divided into multiple intervals and then reassembled to obtain the optimal spectral region for calibration (Additional file 1: Table S3). A principal component analysis (PCA) was carried out to characterize the structure of the spectral population, and the GH outlier (GH  >  3.0) samples were eliminated. Moreover, partial least square (PLS) regression was performed to produce calibration equations. A combination in terms of wavelength range selection and spectrum pretreatment was made to obtain calibration equations in PLS analysis. The internal cross-validation and external validation were carried out to test the performance of the generated equations. The best equations were selected according to the high coefficient of determination of the calibration/internal cross-validation/external validation (*R*^2^/*R*^2^cv/*R*^2^ev), low root mean square error of calibration/internal cross-validation/external validation (RMSEC/RMSECV/RMSEP), and high ratio performance deviation (RPD) values [17, 21]. F-test was applied for comparing the RMSEC/RMSECV values between online and offline NIRS models. *R*^2^ and *R*^2^cv values were converted into continuous variables by Fisher *z*-transformation, and then compared using a Student’s *t* test.

## Supplementary Information

**Additional file 1: Figure S1.** Prediction performance of the obtained equation during integrative online modeling. A-C: Calibration for (A) cellulose crystallinity, (B) lignin clean mass content in dry biomass, and (C) lignin proportion in the cell wall. D-F: Internal cross-validation for (D) cellulose crystallinity, (E) lignin clean mass content in dry biomass, and (F) lignin proportion in the cell wall. ASL, acid-soluble lignin; AIL, acid-insoluble lignin. **Table S1.** Statistics for different collections of sugarcane samples from the NIRS modeling. **Table S2.** Variation in cell wall features in the collected sugarcane population. **Table S3.** Near-infrared spectra pretreatment process for modeling.

## Data Availability

The datasets supporting the conclusions of this article are included within the article and its Additional files.
