# Supplementary material for: Precise high-throughput online near-infrared spectroscopy assay to determine key cell wall features associated with sugarcane bagasse digestibility
Source: Biotechnol Biofuels. 2021 May 29;14:123. doi: 10.1186/s13068-021-01979-x (PMC8164326; doi:10.1186/s13068-021-01979-x)
Supplement: Supplementary file 1 — Additional file 1: Figure S1. Prediction performance of the obtained equation during integrative online modeling. A-C: Calibration for (A) cellulose crystallinity, (B) lignin clean mass content in dry biomass, and (C) lignin proportion in the cell wall. D-F: Internal cross-validation for (D) cellulose crystallinity, (E) lignin clean mass content in dry biomass, and (F) lignin proportion in the cell wall. ASL, acid-soluble lignin; AIL, acid-insoluble lignin. Table S1. Statistics for different collections of sugarcane samples from the NIRS modeling. Table S2. Variation in cell wall features in the collected sugarcane population. Table S3. Near-infrared spectra pretreatment process for modeling. [file 13068_2021_1979_MOESM1_ESM.pdf]

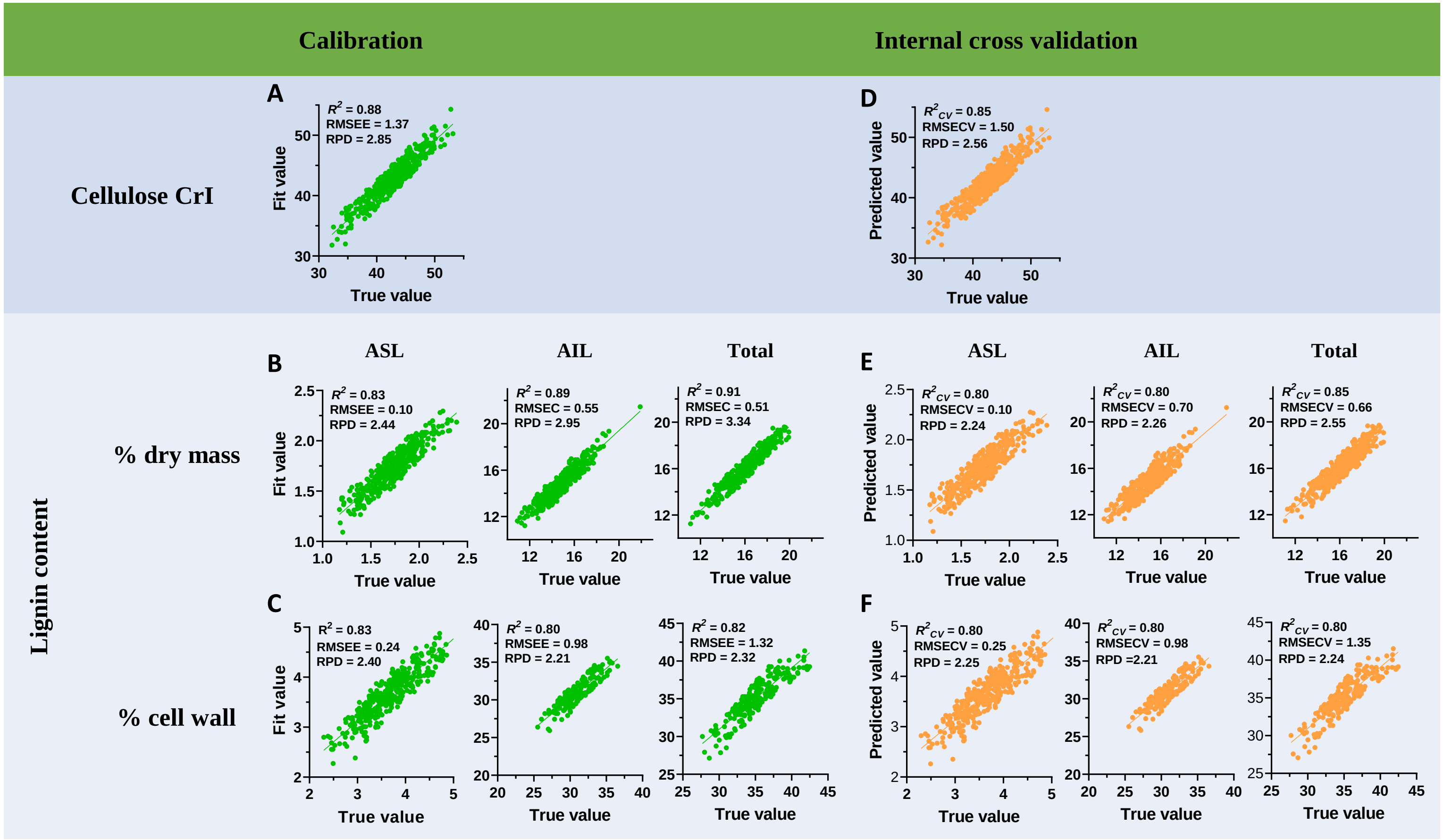

Fig. S1 Equations' prediction performance in integrative online modeling. A-C: Calibration for (A) Cellulose crystallinity; **(B)** Lignin clean mass content in dry biomass; **(C)** Lignin proportion in cell wall**. D-F: Internal cross validation for (D)** Cellulose crystallinity; **(E)** Lignin clean mass content in dry biomass; **(F)** Lignin proportion in cell wall**. ASL**, acid soluble lignin; **AIL,** acid insoluble lignin.

Table S1 Statistic for different collections of sugarcane samples in NIRS modeling.

**Collections**	**Sampling date**	**Counts of genotypes**	**Ripening stages**	**Counts of sugarcane internodes numbers**
**1**	2018.11	164	Pre-maturity	18-20
**2**	2018.12	162	Maturity 1	19-22
**3**	2019.01	184	Maturity 2	19-23
**4**	2019.02	70	Maturity 3	21-24
**5**	2019.03	48	Maturity 4	23-26
**6**	2019.12	210	Additional	18-21

Table S2 Variation of cell wall features in collected sugarcane collections.

	N	Min	Max	Mean	SD	CV
**Cellulose Crl**	838	21.63	55.59	42.70	4.38	0.10
**Lignin content (% dry mass)**						
ASL	679	1.17	2.62	1.76	0.24	0.14
AIL	679	9.16	25.28	14.31	2.07	0.14
Total	679	10.85	27.00	16.08	1.98	0.12
**Lignin proportion in cell wall (%)**						
ASL	563	2.29	7.98	3.71	0.70	0.19
AIL	563	20.20	51.04	31.14	4.26	0.14
Total	563	24.29	56.22	34.87	4.32	0.12

N, sample number; Min, minimum value; Max, maximum value; SD, standard deviation; CV, coefficient of variation; ASL, acid soluble lignin; AIL, acid insoluble lignin.

Table S3 Near-infrared spectra pretreated processing for modeling.

**Scatter correction methods**	**Intrenal of spectrum range (cm^{-1})**									
COE	111987~11198	111987~10409	111987~9620	111987~8830	111987~8041	111987~7252	111987~6463	111987~5674	111987~4885	111987~4096
SSL	11198~10409	11198~9620	11198~8830	11198~8041	11198~7252	11198~6463	11198~5674	11198~4885	11198~4096	
SNV	10409~9620	10409~8830	10409~8041	10409~7252	10409~6463	10409~5674	10409~4885	10409~4096		
MMN	9620~8830	9620~8041	9620~7252	9620~6463	9620~5674	9620~4885	9620~4096			
MSC	8830~8041	8830~7252	8830~6463	8830~5674	8830~4885	8830~4096				
FD	8041~7252	8041~6463	8041~5674	8041~4885	8041~4096					
SED	7252~6463	7252~5674	7252~4885	7252~4096						
FD+SSL	6463~5674	6463~4885	6463~4096							
FD+SNV	5674~4885	5674~4096								
FD+MSC	4885~4096									
	9400~7500	9400~6100	9400~5450	9400~4600	9400~4250					
	7500~6100	7500~5450	7500~4600	7500~4250						
	6100~5450	6100~4600	6100~4250							
	5450~4600	5450~4250								
	4600~4250									

COE, constant offset elimination, SSL, straight line subtraction; SNV, standard normal variate; MMN, Min-Max normalization; MSC, multiplicative scattering correction; FD, first derivative; SED, second derivative; FD+SSL, a combinations of first derivative and straight line subtraction; FD+SNV, a combinations of first derivative and standard normal variate; FD+MSC, a combination of first derivative and multiplicative scattering correction.
